# Hospitalizations and adverse drug events in the Brazilian unified health system: a ten-year retrospective analysis of routine data

**DOI:** 10.11606/s1518-8787.2022056003913

**Published:** 2022-09-30

**Authors:** Lunara Teles Silva, Ana Carolina Figueiredo Modesto, Rodrigo Alves de Oliveira, Rita Goreti Amaral, Flavio Marques Lopes

**Affiliations:** I Universidade Federal de Goiás Faculdade de Medicina Programa de Pós-Graduação em Ciências da Saúde Goiânia GO Brasil Universidade Federal de Goiás. Faculdade de Medicina. Programa de Pós-Graduação em Ciências da Saúde. Goiânia, GO, Brasil; II Universidade Federal de Goiás Hospital das Clínicas Goiânia GO Brasil Universidade Federal de Goiás. Hospital das Clínicas. Goiânia, GO, Brasil; III Universidade Federal de Goiás Faculdade de Farmácia Goiânia GO Brasil Universidade Federal de Goiás. Faculdade de Farmácia. Goiânia, GO, Brasil

**Keywords:** Drug-related side effects and adverse reactions, Pharmacoepidemiology, Hospitalization, Databases, factual

## Abstract

**OBJECTIVE:**

To describe the frequency and characteristics of hospitalizations for/with adverse drug events in the Brazilian unified health system routine data.

**METHODS:**

Nationwide retrospective study using data obtained from a period of ten years from the Brazil Hospital Information System (SIH-SUS), an administrative database that registers hospitalizations in the unified health system. We selected hospitalizations with primary and/or secondary diagnosis related to adverse drug events according to a list of validated International Classification Disease 10^th^ edition (ICD-10) codes. These events were described according to year, age group, sex, length of hospital stay, mortality, hospital costs, Brazilian geographical region, and category of ICD-10 codes. Crude hospitalization rates of adverse drug events per 100,000 inhabitants were obtained and Joinpoint Regression was used to analyze temporal changes in these rates along the years. The most frequent ICD-10 codes were also identified.

**RESULTS:**

Over ten years, 603,663 hospitalizations in Brazil were found in the database, out of which 2.5% of the patients died. Though 2009 had the highest prevalence of hospitalization per 100,000 inhabitants (32.57), no significant annual change in rates was found for the entire period. All age groups and sexes presented a jointpoint in temporal series; however, only women had a significative increase trend. The most frequent codes were from the chapter of mental and behavioral disorders (F19.2, F19.0, and F19.5 codes).

**CONCLUSIONS:**

The database methodology can be useful to estimate frequencies of adverse drug events and perform characterization nationwide and to help monitor morbidity along the years.

## INTRODUCTION

Adverse drug events (ADE) have been considered as a significant cause of hospital admission or complications during hospital stay^1^. ADE are estimated to occur in a mean of 21.6% of patients during hospitalization^4^. In Germany, around 5.0% of hospital admissions were related to ADE over five years^5^. ADE are also associated with increased length of hospital stay (LOS) and risk of death^2 , 6^. A study conducted at a university hospital found that length of stay increased in 1.13 days when an ADE was present^7^. Another study estimated a 10.5% drug-related mortality in a hospital in Spain^3^. ADE also have a great economic burden – ADE-related hospitalization in the USA from 2008 to 2011 totaled $142 billion^2^.

Assessing ADE occurrence nationwide remains a challenge despite the impact of these injuries because of the difficulties of assessment in a large population, the different strategies available for event reporting, incomplete information, or underreporting^1 , 8^. Administrative hospital data, which use International Classification of Diseases (ICD) codes, are being increasingly approached to assess ADE as a potential strategy to complement existing methods for ADE identification^2 , 5^. Studies have found that ICD codes can reasonably detect ADE in hospital routine data^9 , 10^. However, research on ADE using ICD codes varies regarding which codes to use. In Brazil, Mota et al.^11^ validated a list of codes stratified by groups according to their probability of indicating a real ADE. To our knowledge, however, the list has not been applied to analyze hospitalizations related to ADE. This study thus aimed to describe the frequency and characteristics of hospitalizations for/with ADE in the Brazilian unified health system routine data.

## METHODS

### Study Design and Database

A retrospective study was conducted to describe ADE using data from the *Sistema de Informação Hospitalar* (SIH-SUS – Hospital Information System), an administrative database that registers hospitalizations in the Brazilian unified health system (used by more than 70% of the population) since 1992 and records a mean of 11 million registries per year for reimbursement. The database is maintained by the *Departamento de Informática do Sistema Único de Saúde* (DATASUS – Information Technology Department of the Brazilian Unified Health System), a service of the Ministry of Health.

The SIH-SUS collects information from the *Autorização de Internação Hospitalar* (AIH – Inpatient Hospital Authorization), which describes some characteristics of the hospital stay. AIH dataset is available for download and contains patients’ socioeconomic data (age, sex, and race) and clinical characteristics (LOS, costs incurred, and type of admission). It also includes information of patients’ primary and secondary diagnoses using the ICD-10 codes. Data from AIH are registered by coders in each public hospital after discharge and then sent to authorities. When a patient is admitted, an AIH number is generated for the new hospitalization, representing a line in the dataset. If the patient’s length of stay is prolongated, the hospital creates another AIH with the same number of the original. In this case, a new line is added in the dataset and the type of hospitalization changes from new AIH to prolongation AIH^12^.

### Definitions

ADE is defined as “harm experienced by a patient as a result of exposure to a drug” and it involves, for example, anaphylaxis to penicillin or major hemorrhage from overdose of heparin^13^. This study considered two main categories as ADE: (1) adverse drug reaction (ADR) and (2) drug poisoning. ADR are classically defined as harms caused by the use of a drug in a recommended manner, being usually described in ICD as “adverse effects” or with the terms “due to a drug”, “drug-induced”, and “secondary to drugs”^13 , 14^. Vaccines were not included in this category. Drug poisoning was considered as unintentional or undetermined cause of poisoning or toxic effect by medication^14^. Hospitalizations with codes of intentional poisoning as the external cause were not included despite their other codes. “ADE” was the preferable term used in this paper and “hospitalization” was chosen for ADE related to hospital admissions and/or ADE occurring during inpatient treatment.

### ADE Identifications and Data Processing

Hospitalization data were collected from a period of ten years (2009 to 2018) from SIH-SUS, in which hospitalizations with at least one ICD-10 code related to ADE as primary and/or secondary diagnosis were identified as cases. Cases in this study thus include hospitalization that ends in discharge, death, or change in number of the AIH with the ICD-10 codes predefined^11^. If a unique hospitalization had more than one line, data was gathered in a single line. If a patient identified more than one code, their hospitalization was counted only once.

In total, 618 ICD-10 codes were extracted and classified according a list previously validated by Mota et al.^11^ The classification divides the ICD-10 codes into five categories based on the validity of each one as an indicator of event: Certain (A1 – adverse reaction caused by drug and B1 – poisoning by drugs), Probable (A2 – adverse reaction caused by drug or other causes and B2 – poisoning caused by drug or other causes), Possible (C – adverse event possibly expected by drug), and Occasional (D – adverse event occasionally expected by drug).^11^ Supporting information summarizes the codes and categories.

### Variables

The main outcome measure was the detection of ADE related to hospitalizations identified by ICD-10 codes. Secondary outcomes were: year of hospitalization, age group (≤ 4; 5–14; 15–24; 25–34; 35–44; 45–54; 55–64; 65–74; ≥ 75), sex (female and male), LOS, death in hospital, costs of the AIH (in dollars, costs in Brazilian reais were converted to US dollar before the publication of the dataset), geographical region, level of service complexity (moderate or high), and ICD-10 code category (A1/A2/B1/B2/C/D).

### Statistical Analysis

The data were managed and analyzed with software R 3.6.3 (The R Foundation for Statistical Computing, 2019). Overall frequencies of hospitalizations with ADE were calculated by year, age group, sex, deaths, level of complexity, and geographical region. Median costs of the AIH and LOS were calculated overall and by year. Mean/median and standard deviation/interquartile range were computed when possible.

The annual prevalence of hospitalizations (per 100,000 inhabitants) was estimated overall and by sex, age group, and region of Brazil. The number of hospitalizations with at least one ADE code was used as the numerator whereas the population obtained from a Brazilian census projection was the denominator. Temporal trends in rates in the studied period were estimated using the Joinpoint Regression Program Version 4.8.0.1 (Statistical Research and Applications Branch, National Cancer Institute). This method identifies trend changes and describes the joinpoint model using annual percentage change (APC) during a particular trend and average annual percentage change (AAPC) for the entire period^15^.

The ADE frequencies were estimated by each ICD-10 code category regarding sex, age, age group, LOS, AIH costs, and deaths. Since some hospitalizations were registered with two or more ICD-10 codes included in different categories (A1/A2/B1/B2/C/D), the number of hospitalizations with at least one ADE was lower than the sum of identified episodes by each ICD category. The most frequent ICD-10 codes by year, sex, age group, and geographical region were identified. Moreover, ICD-10 codes from categories A1 and B1 were categorized by drug classes and costs of the AIH were studied.

### Ethical Aspects

Data used in this study are public and accessible by all those interested. Nevertheless, the study was approved by the Research Ethics Committee of the *Universidade Federal de Goiás* - UFG (CAEE 32355720.9.0000.5083).

## RESULTS

### Overall and Ten-Year Trends in Hospitalizations Related to ADE

From 2009 to 2018, 607,663 hospitalizations had at least one ICD-10 related to ADE in the Brazilian unified health system. The mean of hospitalizations by year was 60,280.8. Table 1 summarizes the baseline characteristics of the study population. The median age of all patients was 31.0 (IQT: 22.0–44.0) and 33.9% of the patients were women. By age group, was observed in patients aged 25 to 34 years had the highest number of adverse events. Patients were hospitalized for more than 11 days in 39.9% of the cases. ADE accounted for 9,558,623 hospital-bed days during the studied period. The median of health care cost of hospitalizations per year was US$ 25,995,036. In total, 15,486 (2.5%) patients died during hospitalization. Regarding discharge status, most patients were discharged after improving and 6.6% were discharged at their own request.

**Table 1:** Characteristics of the hospitalizations with ICD-10 codes related to adverse drug event in the Brazilian Unified Health System.

Variable	2009	2010	2011	2012	2013	2014	2015	2016	2017	2018	All years
Number of hospitalizations	63,044	62,373	61,658	61,836	59,152	57,971	56,508	56,472	61,818	66,831	607,663
Sex n (%)											
Female	20,187 (32.0)	19,818 (31.8)	19,995 (32.4)	20,027 (32.4)	19,582 (33.1)	19,988 (34.5)	19,881 (35.2)	20,177 (35.7)	22,192 (35.9)	24,351 (36.4)	206,198 (33.9)
Male	42,857 (68.0)	42,555 (68.2)	41,663 (67.6)	41,809 (67.6)	39,570 (66.9)	37,983 (65.5)	36,627 (64.8)	36,295 (64.3)	39,626 (64.1)	42,480 (63.6)	401,465 (66.1)
Age, years m (IQT)	30.0 (22.0–43.0)	30.0 (21.0–42.0)	30.0 (22.0–42.0)	30.0 (22.0–42.0)	31.0 (22.0–43.0)	32.0 (22.0–44.0)	33.0 (23.0–46.0)	33.0 (22.0–47.0)	33.0 (22.0–46.0)	33.0 (22.0–46.0)	31.0 (22.0–44.0)
Age group, years n (%)											
≤ 4	3,062 (4.8)	3,089 (4.9)	2,842 (4.6)	2,911 (4.7)	2,880 (4.8)	2,810 (4.8)	2,843 (5.0)	2,997 (5.3)	3,258 (5.2)	3,349 (5.0)	30,041 (4.9)
5–14	3,021 (4.8)	3,068 (4.9)	3,049 (4.9)	2,801 (4.5)	2,614 (4.4)	2,599 (4.5)	2,558 (4.5)	2,551 (4.5)	2,865 (4.6)	3,043 (4.6)	28,169 (4.6)
15–24	15,046 (23.9)	14,973 (24.0)	14,520 (23.5)	14,359 (23.2)	13,102 (22.2)	12,238 (21.1)	10,932 (19.3)	11,190 (19.8)	12,653 (20.6)	13,555 (20.3)	132,568 (21.8)
25–34	17,475 (27.8)	17,504 (28.2)	17,637 (28.6)	17,585 (28.5)	16,671 (28.3)	15,678 (27.1)	14,372 (25.5)	13,717 (24.3)	14,797 (24.0)	15,861 (23.7)	161,297 (26.6)
35–44	10,098 (16.0)	9,985 (16.0)	10,206 (16.6)	10,599 (17.1)	10,429 (17.6)	10,558 (18.2)	10,427 (18.5)	10,103 (17.9)	11,310 (18.3)	12,735 (19.1)	106,450 (17.5)
45–54	6,017 (9.5)	5,897 (9.5)	5,951 (9.7)	6,102 (9.9)	5,881 (9.9)	5,907 (10.2)	6,149 (10.9)	6,444 (11.4)	6,702 (10.8)	7,430 (11.1)	62,480 (10.3)
55–64	3,448 (5.5)	3,455 (5.5)	3,319 (5.4)	3,316 (5.4)	3,346 (5.7)	3,650 (6.3)	4,005 (7.1)	4,215 (7.5)	4,414 (7.1)	4,900 (7.3)	38,068 (6.3)
65–74	2,606 (4.1)	2,385 (3.8)	2,165 (3.5)	2,193 (3.5)	2,312 (3.9)	2,364 (4.1)	2,713 (4.8)	2,790 (4.9)	3,153 (5.1)	3,252 (4.9)	25,933 (4.3)
≥ 75	2,271 (3.6)	2,017 (3.2)	1,969 (3.2)	1,970 (3.2)	1,917 (3.2)	2,167 (3.7)	2,509 (4.4)	2,465 (4.4)	2,666 (4.3)	2,706 (4.0)	22,657 (3.7)
LOS, days m ( IQT)	6.0 (3.0–19.0)	7.0 (3.0–20.0)	7.0 (3.0–20.0)	7.0 (3.0–21.0)	7.0 (2.0–21.0)	7.0 (2.0–20.0)	6.0 (2.0–20.0)	6.0 (2.0–19.0)	6.0 (2.0–19.0)	7.0 (2.0–19.0)	7.0 (3.0–20.0)
Hospitalization costs, Brazilian Real m (IQT)	367.44 (160.95–849.40)	407.17 (184.79–1,041.98)	420.00 (184.79–1,080.57)	438.68 (184.87–1,120.00)	445.79 (183.72–1,140.00)	416.39 (169.48–1,140.00)	416.39 (171.00–1,140.00)	426.10 (177.14–1,140.00)	439.52 (184.87–1,140.00)	456.00 (177.35–1,197.00)	416.4 (173.90–1,104.20)
Hospitalization costs, US dollar m (IQT)	191.22 (85.74–450.62)	235.80 (102.79–598.80)	251.78 (106.42–639.10)	219.83 (91.20–560.35)	198.80 (80.28–511.53)	173.05 (69.76–479.20)	117.19 (48.61–317.59)	128.45 (52.59–347.11)	136.78 (56.53–354.80)	122.47 (47.77–321.05)	173.95 (74.54–445.45)
Inpatient deaths n (%)											
Yes	1,294 (2.1)	1,271 (2.0)	1,282 (2.1)	1,278 (2.1)	1,349 (2.3)	1,341 (2.3)	1,685 (3.0)	1,907 (3.4)	2,009 (3.2)	2,070 (3.1)	15,486 (2.5)
No	61,750 (97.9)	61,102 (98.0)	60,376 (97.9)	60,558 (97.9)	57,803 (97.7)	56,630 (97.7)	54,823 (97.0)	54,565 (96.6)	59,809 (96.8)	64,761 (96.9)	592,177 (97.5)
Level of complexity											
Moderate	62,399 (99.0)	61,660 (98.9)	60,990 (98.9)	61,248 (99.0)	58,562 (99.0)	57,338 (98.9)	55,792 (98.7)	55,654 (98.6)	60,760 (98.3)	65,878 (98.6)	600,281 (98.8)
High	645 (1.0)	713 (1.1)	668 (1.1)	588 (1.0)	590 (1.0)	633 (1.1)	716 (1.3)	818 (1.4)	1,058 (1.7)	953 (1.4)	7,382 (1.2)
Intensive care											
Yes	2,471 (3.9)	2,514 (4.0)	2,419 (3.9)	2,514 (4.1)	2,570 (4.3)	2,666 (4.6)	2,946 (5.2)	3,296 (5.8)	3,603 (5.8)	3,914 (5.9)	28,913 (4.8)
No	60,573 (96.1)	59,859 (96.0)	59,239 (96.1)	59,322 (95.9)	56,582 (95.7)	55,305 (95.4)	53,562 (94.8)	53,176 (94.2)	58,215 (94.2)	62,917 (94.1)	578,750 (95.2)
Brazilian regions											
North	2,080 (3.2)	2,034 (3.2)	1,947 (3.1)	2,139 (3.5)	2,493 (4.1)	2,567 (4.4)	2,321 (4.1)	2,197 (3.9)	2,235 (3.6)	2,309 (3.4)	22,322 (3.7)
Northeast	10,630 (16.9)	10,085 (16.2)	9,119 (14.8)	8,941 (14.5)	8,939 (15.1)	8,929 (15.4)	8,819 (15.6)	8,819 (15.6)	9,064 (14.7)	9,328 (14.0)	92,673 (15.3)
Midwest	6,447 (10.2)	5,584 (9.0)	5,728 (9.3)	6,145 (9.9)	5,254 (8.9)	4,247 (7.3)	4,130 (7.3)	3,827 (6.7)	4,295 (6.9)	4,911 (7.3)	50,568 (8.3)
Southeast	27,271 (43.3)	27,887 (44.7)	28,523 (46.3)	28,967 (46.8)	28,228 (47.8)	27,324 (47.2)	25,901 (45.9)	25,735 (45.7)	27,604 (44.7)	29,994 (44.9)	277,434 (45.7)
South	16,616 (26.4)	16,783 (26.9)	16,341 (26.5)	15,644 (25.3)	14,238 (24.1)	14,904 (25.7)	15,337 (27.1)	15,894 (28.1)	18,620 (30.1)	20,289 (30.4)	164,666 (27.0)

ICD-10: International Classification of Disease 10^th^ edition; LOS: length of hospital stay; m: mean; IQT: interquartile range.

Table 2 describes crude rates of hospitalizations related to ADE per 100,000 inhabitants in Brazil and the trends in rates according to the joinpoints (i.e., a trend change). During the studied period, the highest rate of hospitalization for ADE was registered in Brazil in 2009 (32.57 per 100,000 inhabitants). Men and the age group 25 to 34 years old presented higher rates of ADE. A joinpoint was identified in rates by overall hospitalizations in 2016, leading to two periods with different trends: a decrease followed by an increase. The same occurred between the rates in women and men, but only women had a significant annual increase (1.4%) during the period. All age groups presented a joinpoint in time series; however, only segments represented by APC were significant. No joinpoints were found in the analysis of rates according to Brazilian regions, that is, APC equals AAPC, and only two regions showed a significant decrease in rates. The inpatient mortality rate related to ADE per 100,000 inhabitants showed a joinpoint in 2013, with 9.8% increase from 2013 to 2018 (data not shown in Table 2 ).

**Table 2:** Hospitalization in the Brazilian Unified Health System with ICD-10 codes of adverse drug event per 100,000 inhabitants (crude rates).

	2009	2010	2011	2012	2013	2014	2015	2016	2017	2018	APC (95%CI)	AAPC (95%CI)
All hospitalizations	32.57	32.00	31.36	31.18	29.58	28.74	27.77	27.53	29.89	32.05	2009–2016: -2.6^a^ (-3.2 to -1.9)	-0.3 (-1.2 to 0.7)
											2016–2018: 8.2^a^ (3.1 to 13.7)	
Hospitalizations by sex												
Female	20.65	19.94	19.94	19.79	19.18	19.41	19.14	19.26	21.01	22.86	2009–2016: -0.9^a^ (-1.5 to -0.4)	1.4^a^ (0.6 to 2.1)
											2016–2018: 9.8^a^ (5.4 to 14.3)	
Male	44.75	44.55	43.25	43.04	40.41	38.47	36.78	36.16	39.48	41.66	2009–2016: -3.3^a^ (-4.4 to -2.2)	-1.0 (-2.5 to 0.5)
											2016–2018: 7.5 (-1.0 to 16.7)	
Hospitalizations by age group												
≤ 4 years	19.08	20.79	19.34	19.91	19.77	19.29	19.37	20.37	22.13	22.65	2009–2015: -0.3 (-2.3 to 1.7)	1.6 (-0.2 to 3.5)
											2015–2018: 5.5 (-0.6 to 12.0)	
5–14 years	8.82	9.22	9.29	8.68	8.25	8.34	8.34	8.44	9.56	10.24	2009–2015: -2.0 (-4.1 to 0.2)	1.1 (-0.8 to 3.1)
											2015–2018: 7.6^a^ (1.0 to 14.7)	
15–24 years	43.40	43.15	41.92	41.49	37.87	35.42	31.75	32.64	37.19	40.18	2009–2016: -4.7^a^ (-6.7 to -2.6)	-1.2 (-4.1 to 1.8)
											2016–2018: 12.2 (-4.4 to 31.6)	
25–34 years	52.49	52.58	52.34	51.69	48.66	45.55	41.65	39.73	42.93	46.15	2009–2016: -4.2^a^ (-6.2 to -2.2)	-2.1 (-4.9 to 0.8)
											2016–2018: 5.7 (-9.4 to 23.3)	
35–44 years	37.67	36.63	36.90	37.71	36.48	36.27	35.14	33.38	36.60	40.38	2009–2016: -1.4^a^ (-2.2 to -0.6)	0.6 (-0.6 to 1.7)
											2016–2018: 7.8^a^ (1.4 to 14.7)	
45–54 years	27.80	26.48	26.11	26.20	24.74	24.40	25.00	25.82	26.52	29.05	2009–2014: -2.6^a^ (-4.2 to -1.0)	0.3 (-0.8 to 1.3)
											2014–2018: 4.0^a^ (1.6 to 6.5)	
55–64 years	24.61	23.06	21.36	20.60	20.09	21.19	22.49	22.93	23.27	25.07	2009–2012: -6.7^a^ (-9.4 to -3.9)	-0.0 (-0.9 to 0.9)
											2012–2018: 3.5^a^ (2.5 to 4.5)	
65–74 years	33.02	27.42	24.10	23.60	24.01	23.65	26.10	25.78	27.95	27.65	2009–2011: -16.2^a^ (-24.5 to -6.9)	-1.9 (-3.8 to 0.0)
											2011–2018: 2.6^a^ (1.2 to 4.0)	
≥ 75 years	46.29	36.19	34.09	32.83	30.73	33.44	37.35	35.43	37.00	36.25	2009–2011: -15.7 (-29.6 to 0.8)	-2.2 (-5.4 to 1.2)
											2011–2018: 2.1 (-0.3 to 4.6)	
Hospitalizations by Brazilian Regions												
North	13.05	12.52	11.81	12.78	14.69	14.91	13.29	12.42	12.47	12.70	2009–2018: 0.1 (-1.9 to 2.1)	0.1 (-1.9 to 2.1)
Northeast	19.67	18.60	16.72	16.29	16.20	16.09	15.80	15.71	16.06	16.43	2009–2018: -1.8^a^ (-3.1 to -0.5)	-1.8^a^ (-3.1 to -0.5)
Midwest	45.87	38.91	39.32	41.57	35.04	27.91	26.74	24.44	27.06	30.53	2009–2018: -6.0^a^ (-8.8 to -3.0)	-6.0^a^ (-8.8 to -3.0)
Southeast	33.40	33.94	34.43	34.67	33.50	32.16	30.23	29.80	31.72	34.20	2009–2018: -0.9 (-2.1 to 0.4)	-0.9 (-2.1 to 0.4)
South	59.64	60.11	58.06	55.15	49.79	51.70	52.76	54.25	63.06	68.19	2009–2018: 0.7 (-1.9 to 3.3)	0.7 (-1.9 to 3.3)

ICD-10: International Classification Disease 10^th^ edition; 95%CI: 95% confidence interval.

^a^ Indicates that the annual percent change (APC) or average annual percentage change (AAPC) is significantly different from zero at the alpha = 0.05 level.

### Overall Description of the ICD-10 Codes

In total, 638,685 ICD-10 codes were assigned to hospitalizations related to ADE in the Brazilian unified health system. Codes from the category “certain” (A1 and B1) were found in 90,452 (14.9%) hospitalizations. Cases from this category accounted for 404,821 hospital-bed days. Around 9.3% of all costs were related to hospitalizations with codes of the category “certain”. Codes from the category “probable” (A2 and B2) appeared in 70.6% of cases ( Table 3 ). Supporting information has the frequencies of hospitalizations with each ICD-10 code.

**Table 3:** Distribution of hospitalizations in the Brazilian Unified Health System related to adverse drug event by ICD-10 codes category (A1/A2/B1/B2/C/D).

Description of codes category	Number of codes	Number of hospitalizations^a^	Female n (%)	Age m (IQT)	Age group n (%)	LOS (total)	LOS m (IQT)	Costs (total)	Costs m (IQT)	Intensive care n (%)	Deaths n (%)
Certain (A1 e B1)	407	90,452	53,576 (59.2)	32.0 (19.0–50.0)	15–24 17,378 (19.2)	404,821	2.0 (2.0–4.0)	59,727,310 (Real)^b^	197.1 (136.9–366.0)	8,432 (9.3)	2,441 (2.7)
								24,150,518 (US$)	81.51 (57.78–148.73)		
Probable (A2 e B2)	170	429,101	114,218 (26.6)	30.0 (22.0–39.0)	25–34 136,521 (31.8)	8,462,648	10.0 (3.0–25.0)	463,955,617 (Real)^b^	546.7 (171.0–1,205.4)	12,839 (3.0)	4,411 (1.0)
								193,135,101 (US$)	221.44 (76.79–512.32)		
Possible (C)	18	16,911	7,366 (43.6)	48.0 (29.0–62.0)	45–54 2,996 (17.7)	150,830	6.0 (3.0–11.0)	22,100,551 (Real)^b^	488.75 (416.39–821.62)	1,461 (8.6)	2,132 (12.6)
								8,101,549 (US$)	213.53 (141.06–333.55)		
Occasional (D)	23	81,282	37,225 (45.8)	47.0 (24.0–64.0)	55–64 12,679 (15.6)	570,988	4.0 (2.0–8.0)	92,161,986 (Real)^b^	354.68 (210.06–717.97)	7,312 (9.0)	6,750 (8.3)
								36,127,441 (US$)	143.43 (88.67–296.22)		

ICD-10: International Classification of Disease 10^th^ edition; LOS: length of hospital stay; m (IQT): mean interquartile range.

^a^ A number of hospitalizations with at least one code from the category.

^b ^Brazilian Real.

Note: A1: adverse reaction caused by drugs; A2: adverse reaction caused by drugs or other causes; B1: poisoning caused by drugs; B2: poisoning caused by drugs or other causes; C: adverse drug event possibly expected for a drug; D: adverse drug event occasionally expected for a drug.

The three most common ICD-10 codes in numbers were those from chapter V (mental and behavioral disorders), F19.2 (187,130; 29.3%), F19.0 (45,635; 7.1%), and F19.5 (30,756; 4.8%), followed by the code of poisoning by unspecified substance T50.9 (27,873; 4.3%) and the code D70 (agranulocytosis) (23,813; 3.7%). Figure A shows the ten codes most related to ADE in each of the ten years analyzed. These codes represent more than 57% of all events in each age group. Figure B shows the distribution of the most prevalent codes except for F19.2, which had higher prevalence (43.5; 50.0%) than other principal codes in all years studied. Codes from chapter V appeared at least five times among the first ten most frequent codes in all years.

**Figure:**
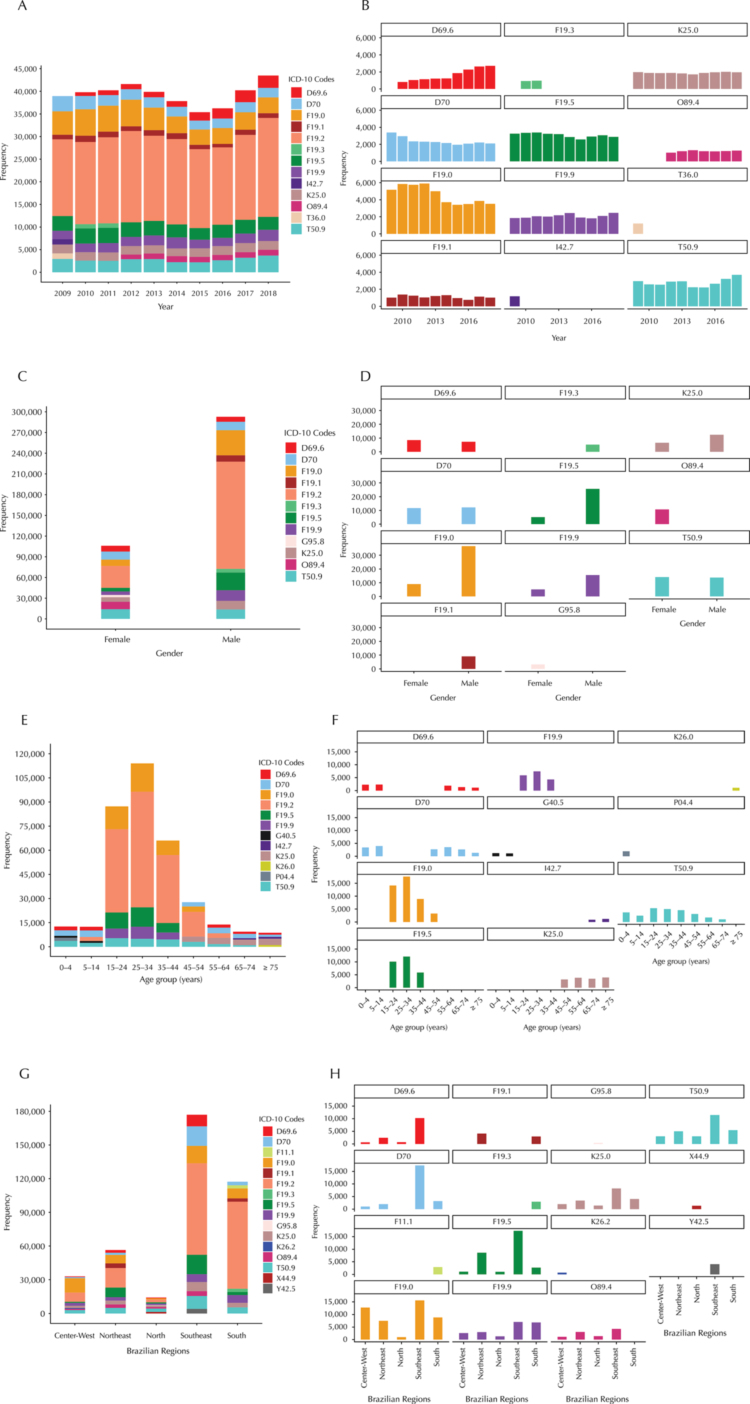
Most frequent ICD-10 codes related to hospitalization and adverse drug events in the Brazilian unified health system by year, sex, age group and geographical region. Continue

D69.6 Thrombocytopenia, unspecified; D70 Agranulocytosis; F11.1 Mental and behavioral disorders due to use of opioids, harmful use; F19.0 Mental and behavioral disorders due to multiple drug use and use of other psychoactive substances, acute intoxication; F19.1 Mental and behavioral disorders due to multiple drug use and use of other psychoactive substances, harmful use; F19.2 Mental and behavioral disorders due to multiple drug use and use of other psychoactive substances, dependence syndrome; F19.3 Mental and behavioral disorders due to multiple drug use and use of other psychoactive substances, withdrawal state; F19.5 Mental and behavioral disorders due to multiple drug use and use of other psychoactive substances, psychotic disorder; F19.9 Mental and behavioral disorders due to multiple drug use and use of other psychoactive substances, unspecified; G40.5 Special epilepsy syndromes; G95.8 Other specified diseases of spinal cord; I42.7 Cardiomyopathy due to drugs and other external agents; K25.0 Gastric ulcer: acute with hemorrhage; K26.0 Duodenal ulcer: acute with hemorrhage; K26.2 Duodenal ulcer: acute with hemorrhage and perforation; O89.4 Spinal and epidural anesthesia-induced headache during the puerperium; P04.4 Fetus and newborn affected by maternal use of drugs of addiction; T36.0 Poisoning by Penicillins; T50.9 Other and unspecified drugs, medicaments and biological substances; X44.9 accidental poisoning by exposure to other and unspecified drugs, medications, and biological substances; Y42.5 Drugs, medications, and biological substances with adverse effects in therapeutic use, other estrogens and progestogens

The database analysis identified 97,604 ICD-10 codes from category “certain”. The main codes from category A1 were O89.4 (spinal and epidural anesthesia-induced headache during the puerperium) (10,724; 1.7%), Y42.5 (events by estrogens and progestogens) (4,052; 0.6%), and Y57.8 (events by other drugs and medicaments) (3,895; 0.6%). In category B1, the most poisonings by unintentional or undetermined cause were T42.4 (benzodiazepines) (4,632; 0.7%), T36.9 (systemic antibiotic, unspecified) (4,236; 0.7%), and T45.5 (anticoagulants) (3,543; 0.6%).

The ten most frequent codes between sexes were almost equal except for O89.4 (spinal and epidural anesthesia-induced headache during the puerperium) and G95.8 (other specified diseases of spinal cord), which appeared only in women, and F19.1 (mental and behavioral disorders, harmful use) and F19.3 (mental and behavioral disorders, withdrawal state), which appeared only in men (Figures C and D). The five codes most prevalent among age groups were analyzed. None of the codes was repeated in any age group (Figures E and F). Four codes were most common in the five regions of Brazil: T50.9, K25.0, F19.5, F19.0, and F19.9 (Figures G and H).

Table 4 provides an overview of the most common classes of substances associated with ADE from codes of the category “certain” (A1 and B1). The most frequent drugs from category A1 were anesthetics and therapeutic gases and hormones (including synthetic substitutes and antagonists). In category B1, the two main classes related to drug poisoning were (1) systemic anti-infectives and (2) antiepileptics, antiparkinsonism drugs, sedatives-hypnotics and antianxiety drugs.

**Table 4:** Description of codes related to adverse drug events according to drug class and costs related to hospitalization with at least one code from the class.

Medication group	Category A1		Costs^b^	Category B1		Costs^b^
	
	ICD-10 codes	Frequency^a^		ICD-10 codes	Frequency^a^	
Systemic anti-infectives	Y40, Y41, Z88.0-Z88.3	2,459	814,909.4	T36, T37	10,113	1,879,595
Hormones, synthetic substitutes, and antagonists	Y42	4,485	1,568,293	T38	1,450	309,954.9
Steroids		23	-		80	-
Insulin and hypoglycemics		230	-		411	-
Thyroid hormones and antithyroid agents		40	-		568	-
Ovarian hormones		4,079	-		208	-
Androgens and anabolic congeners		55	-		84	-
Primarily systemic agents	Y43	1,853	953,935.5	-	-	-
Antineoplastic and immunosuppressive drugs		669	-	T45.1	218	***
Antiallergic and antiemetic drugs		110	-	T45.0	410	***
Agents primarily affecting blood constituents	Y44	2,936	581,367.2	-	-	-
Anticoagulants		2,678	-	T45.5-T45.7	4,071	***
Analgesics, antipyretics, and anti-inflammatory agents	Y45, F11.7, N14.0, Z88.6	1,653	547,044.7	T39, T40.2, T40.3, T40.4 X40, Y10	6,260	915,774
Opioid/narcotics		397	-		65	-
Analgesics (nonopioid)		478	-		6,195	-
Antiepileptics, antiparkinsonian drugs, sedatives-hypnotics, and antianxiety drugs	Y46, Y47, Q86.1	1,450	364,334.3	T42	7,636	2,161,903
Antiepileptics		397	-		-	-
Antiparkinsonian		4	-		837	-
Benzodiazepines		671	-		4,632	-
Anesthetics and therapeutic gases	Y48, Z88.4, O29.3, O74.4, O74.5, O89.3, O89.4, P04.0, T88.2, T88.3	12,562	1,242,493	T41	783	175,980.5
Psychotropic drugs, not classified elsewhere	Y49 (except Y49.6), G21.0	1,996	695,304.2	T43	7,460	2,011,832
Antidepressants		540	-		5,845	-
Antipsychotics		1,456	-		16,15	-
Central nervous system stimulants (not classified elsewhere)	Y50	216	43,850.1	-	-	-
Drugs primarily affecting the autonomic nervous system	Y51	858	1,981,289	T44, X43, Y13	3,647	1,164,609
Cholinergic		8	-		386	-
Anticholinergic		60	-		1,266	-
Adrenergic		13	-		252	-
Antiadrenergic		192	-		225	-
Agents primarily affecting the cardiovascular system	Y52	377	146,324.9	T46	1,477	324,338.2
Cardiac glycosides		105	-		311	-
Antihypertensives (diuretics and antiadrenergic drugs not included)		174	-		766	-
Antihyperlipidemic		8	-		22	-
Antiarrhythmic		12	-		378	-
Agents primarily affecting the gastrointestinal system	Y53	511	118,984.9	T47	1,150	173,544
Antacids and antiulcer secretion drugs		85	-		77	-
Laxatives and digestants		72	-		930	-
Antidiarrheal		16	-		143	-
Emetics		15	-		93	-
Agents primarily affecting water balance and mineral and uric acid metabolism	Y54	194	48,432.29	-	-	-
Diuretics		70		T50.0-T50.2	365	****
Uric acid metabolism drugs		29		T50.4	117	****
Agents primarily acting on smooth and skeletal muscles and the respiratory system	Y55	174	43,886.79	T48	557	100,944
Muscle		46	-		157	-
Antitussives, expectorants, and common medications for colds		36	-		302	-
Asthma medications		29	-		98	-
Topical agents primarily affecting skin and mucous membrane and ophthalmological, otorhinolaryngological, and dental drugs	Y56	540	120,397.6	T49	2,785	730,399.2
Other and unspecified drugs and medications	Y57	4,463	897,353.1	-	-	-
Anorectics		93	-	T50.5	73	****

^a^ Frequency of codes.

^b^ Costs of hospitalization with at least one of the codes.

*** T45 (T45.0-T45.9): poisoning by primarily systemic and hematological agents, not classified elsewhere (frequency = 5,246; costs = 1,205,318).

**** T50 (T50.0-T50.2 and T50.4-T50.8, except T50.3 and T50.9 because they are category; B2: poisoning by diuretics and other and unspecified drugs, medications, and biological substances (frequency = 1,119; costs = 291,761.3).

## DISCUSSION

Our study provided a comprehensive, population-level assessment of the impact of adverse drug events in Brazil’s unified health system. From 2009 to 2018, 607,663 hospitalizations were registered with at least one ICD-10 code related to ADE, out of which 2.5% patients died. Our results show that, overall, these rates of hospitalizations per 100,000 inhabitants had a pattern of decrease followed by an increase during the ten years in two temporal segments. Several studies show a yearly increasing trend in the prevalence and rates of ADE-related hospitalizations^2 , 6 , 16 , 17^. Santos and Boing^16^ showed that hospitalizations caused by ADE in Brazil increased 1.6 times over 15 years. Cases of ADE are expected to increase with time considering the number of drugs that enter the market every year, the prescription/use of many drugs, and improvements in database registrations^17^. In recent years, national ADE patterns – as described by our results – have been addressed with greater awareness about patient safety and continuously improving pharmacovigilance practices, as seen with the Third Global Patient Safety Challenge: Medication Without Harm, launched in 2017^18^.

Our results on the frequencies of hospitalizations related to ADE were different from those of other authors. Two studies conducted in a Germany database found higher annual frequencies than our analysis^5 , 19^; one of them identified 775,959 inpatient stays with ICD-10 codes related to ADE in a year^19^. Differences in frequencies and rates among studies can be partially explained by aspects such as the lack of standardization, especially regarding ADE definitions and ICD coding framework. We defined ADE as “any harm experienced as the result of drug exposure” and included ICD-10 codes of “adverse effects” and “poisoning of unintentional/undetermined cause” previously validated for the Brazilian reality for a wide approach of types of drug harm^11^. Other aspects that can contribute to differences between studies are the structure of the population involved – such as a higher presence of older age groups –, differences in prescribing standards, polypharmacy, as well as differences in documentation and database types^19 , 20^.

Codes associated with ADE-related hospitalizations usually vary between countries, although specific codes are common across studies. As an example, the codes T50.9 and D70 appeared both in our study and in a German study as two of the ten most frequent codes found in primary diagnosis^5^. In studies using Brazilian databases, codes related to “other and unspecified drugs” were common in national registries of hospitalizations, in which the most frequent code was T50.9^21 , 22^. On the other hand, in analyses of databases from Germany, England, and the USA, the most frequent ADE was enterocolitis caused by *Clostridium difficile* (code A04.7)^19^. Codes from chapter V (mental and behavioral disorders) were 52% of all codes studied, where F19.2 alone corresponded to 29.3% of the codes in our results. In Brazil, Chile, and England, F19 codes were one of the two main causes of events^16 , 17 , 20 , 23 , 24^.

By analyzing codes from the category “certain” only, we found that about 15% of hospitalizations were related to this category. These codes allow identifying a drug as the only one responsible for ADE, excluding confounding factors^11^. A German study, for example, found less cases of hospitalizations related to ADE with codes A1 and B1 (almost half of what we found)^5^. Moreover, “systemic anti-infectives” and “anesthetics and therapeutic gases” were the two main classes of drugs analyzed from codes of the “certain” category. Similarly to other studies^2 , 25^, our results reported anti-infectives as one of the most prevalent classes – likely because antibiotics are drugs commonly used in inpatient hospitalization. A study from the Johns Hopkins Hospital showed that 20% of hospitalized patients receiving antibiotics for at least one day developed an ADE^26^. Medications are currently not included in hospital routine data although some ICD-10 codes cover such information, including T36-T50 “poisoning” and Y40-Y59 “adverse effects”. Identifying similarities between causative medications could guide further investigation of underlying reasons for particular types of ADE.

During the studied period, registered ADE amounted to US$ 259.9 million in hospitalization costs and 9.5 million hospital-bed days. Considerable costs such as those described are generally associated with hospitalizations related to ADE. In Spain, ADR alone cost health systems around €1.533 billion for over six years^27^. The US, in turn, had US$ 1,851.43 of additional costs from ADE-related hospitalization^2^. The economic burden to the health system is thus related to the allocation of resources for symptoms diagnosis and treatment.

Studies using administrative databases generally report lower ADE frequencies than studies using intensive monitoring and higher frequencies than spontaneous reporting^27 , 28^. In African hospitals, the median of patients with ADE during hospital stay was 8.4% whereas the median of patients admitted for ADE was 2.8% considering studies that used record review, clinical examination, or interview^29^. Nevertheless, large health databases are great sources of real-world data regarding drug safety which allow characterizing ADE nationwide and indicating critical points^17 , 30^. The increasing availability of large databases offers opportunities to investigate several ADE and detect signals as a complement of spontaneous reporting^5^.

Our study provides evidence regarding the use of health system routine data to estimate the main features of hospitalizations for ADE during a decade in Brazil, showing increasing trends in hospitalizations and deaths. The study also showed that ICD-10 codes in the “certain” category of validity (A1 and B1) as ADE indicators were partly responsible for the costs of hospitalizations. Anti-infectives and anesthetics and therapeutic gases were the drugs most frequently involved in cases with “certain” codes, but ICD-10 codes related to unspecified drugs were prevalent overall. While this assessment focuses on frequency and tendency over time, future studies should determine patients at risk of hospitalization related to ADE nationwide for additional policy recommendations.

### Strengths and Limitations

This study has strengths, including the assessment of a period of ten years within a nationwide scope; in fact, one of the advantages of using administrative databases to study ADE is the possibility of efficiently assessing large populations over long periods of time. Moreover, the list of codes applied was previous validated and includes several situations related to drug use. However, the study has limitations. The use of ICD-10 codes and administrative databases, including the SIH-SUS dataset, to identify potential ADE themselves has limitations. Firstly, regarding the database, factors such as incompleteness, misclassification, and variation among codifiers must be emphasized even with coding-quality monitoring. In this study, we could not estimate error caused by biases^23 ,36^. Secondly, regarding ICD-10 codes, we were careful to exclude cases which might be related to drug abuse, use of illicit substance, and intentional overdose; however, we used some codes that include both illicit and licit drugs, which might be confounding.
